# ERalpha-status of disseminated tumour cells in bone marrow of primary breast cancer patients

**DOI:** 10.1186/bcr2143

**Published:** 2008-09-15

**Authors:** Tanja Fehm, Natalia Krawczyk, Erich-Franz Solomayer, Graziella Becker-Pergola, Silke Dürr-Störzer, Hans Neubauer, Harald Seeger, Annette Staebler, Diethelm Wallwiener, Sven Becker

**Affiliations:** 1 Department of Obstetrics and Gynecology, University of Tuebingen, Calwerstrasse 7, D-72076 Tuebingen, Germany; 2 Department of Pathology, University of Tuebingen, Liebermeisterstrasse 8, D-72076 Tuebingen, Germany

## Abstract

**Introduction:**

Isolated disseminated tumour cells (DTC) are regarded as surrogate markers for minimal residual disease in breast cancer. Characterisation of these cells could help understand the known limitations of adjuvant therapy. Of particular interest is their oestrogen-receptor (ER) status because endocrine adjuvant therapy remains a cornerstone of breast cancer treatment.

**Methods:**

Bone marrow (BM) aspirates from 254 patients with primary breast cancer were included in this study. A double immunofluorescence staining procedure was established for the identification of cytokeratin (CK) positive/Erα-positive cells. ERα status of the primary tumour was assessed immunohistochemically using the same antibody against ERα.

**Results:**

In 107 of 254 (42%) breast cancer patients, CK-positive cells could be detected in the BM. More than one DTC in the BM was observed in 38 of the 107 patients. The number of detected cells ranged between 1 and 55 cells per 2 × 10^6 ^mononuclear cells. DTCs demonstrated ERα positivity in 12% of the patients. The ERα expression was heterogeneous in 10 of the 38 (26%) patients with more than one DTC. The concordance rate of ERα status between primary tumour and DTC was 28%. Only 12 of 88 patients with ERα-positive tumours also had ERα-positive DTCs.

**Conclusions:**

Primary tumours and DTCs displayed a concordant ERα status in only 28% of cases. Most of the DTCs were ERα negative despite the presence of an ERα-positive primary tumour. These findings further underline the distinct nature of DTCs and may explain the failure rates seen in conventional endocrine adjuvant therapy.

## Introduction

Tumour cell dissemination is a common phenomenon in breast cancer where isolated disseminated cells can be detected in up to 40% of patients at the time of primary diagnosis [1-3]. Based on the pooled analysis of the bone marrow (BM) micrometastasis group, disseminated tumour cells (DTC) are a surrogate marker of minimal residual disease. Their presence is associated with a poor prognosis [4]. With their prognostic significance clearly demonstrated, efforts have been made to further characterise these cells using pheno- and genotyping techniques. Studies have shown that the persistence of DTCs in the BM of patients with primary breast cancer after conventional adjuvant therapy is associated with a poor prognosis [5-7].

More detailed knowledge about their cellular and molecular characteristics could help define a targeted secondary adjuvant therapy in patients with primary breast cancer who have undergone conventional adjuvant therapy. It has already been shown that about 40% of DTCs express human epidermal growth factor receptor 2 (HER2) and that in some patients with recurrent breast cancer their HER2 status may differ from that of the primary tumour [8]. Since the most widely used form of targeted therapy for breast cancer remains anti-oestrogen endocrine therapy, it is important to know if the ERα status of DTCs corresponds to the ERα status of the primary tumour, particularly in view of the 15 to 20% relapse rate in early stage ERα-positive tumours despite adjuvant endocrine therapy [9]. Furthermore, while ERα-negative tumours are not considered candidates for endocrine therapy, the ERα status of DTCs may differ from the primary tumour. The goal of this study was to determine the ERα status of DTCs in BM of breast cancer patients, and to compare the ERα status of DTCs and the corresponding primary tumours.

## Materials and methods

### Collection and analysis of bone marrow

Prior to any therapy, between 10 and 20 ml of bone marrow were aspirated from the anterior iliac crest of 254 primary breast cancer patients undergoing surgical treatment from 2005 to 2007 at the Department of Gynecology and Obstetrics, University Hospital Tuebingen, Germany.

The characteristics of the patients are shown in Table 1. All specimens were obtained after written informed consent was given and were collected using protocols approved by the institutional review board (114/2006A). Tumour cell isolation and detection was performed based on the recommendations for standardised tumour cell detection [10]. BM samples were separated by density centrifugation over Ficoll with a density of 1.077 g/ml (Biochrom, Germany). If necessary red blood cells were lysed with lysis buffer (155 mM NH_4_Cl, 10 mM KHC0_3_, 0.1 mM EDTA pH 7.2). Using a cytocentrifuge (Hettich, Tuttlingen, Germany), 10^6 ^mononuclear cells were spun onto a glass slide. The slides were air-dried overnight at room temperature. For detection and characterisation of DTCs, slides were fixed in a 0.5% neutral buffered formalin solution for 10 minutes. Control cytospins with ERα-positive MCF-7 cells were prepared, stored and fixed in the same way to ensure that ERα negativity of a patient's sample was not due to a handling error. Two slides per patient was analysed for the presence of DTCs (2 × 10^6 ^cells per patient).

**Table 1:** Clinical data of patients

	n = 254	BM positive (%)	p-value*
Total	254	107 (42)	
Menopausal status			
Premenopausal	79	33 (42)	0.94
Postmenopausal	175	74 (42)	
Tumour size			
pT1	148	60 (41)	0.77
pT2-4	103	46 (45)	
Nodal status			
Node negative	149	62 (42)	0.88
Node positive	101	43 (43)	
Histology			
Ductal	177	74 (42)	0.12
Lobular	57	21 (37)	
Others	17	11 (65)	
Grading			
I to II	217	91 (42)	0.85
III	32	14 (44)	
ER status			
Negative	42	19 (45)	0.83
Positive	208	88 (42)	
PR status			
Negative	69	26 (38)	0.39
Positive	181	79 (44)	
HER2			
Negative (0/+1)	211	94 (45)	0.08
Positive (+2/+3)	32	9 (28)	

* by Chi-squared test. BM = bone marrow; ER = oestrogen receptor; HER2 = human epidermal growth factor receptor 2; PR = progesterone receptor.

### Optimising the ERα staining protocol

**Figure 1:**
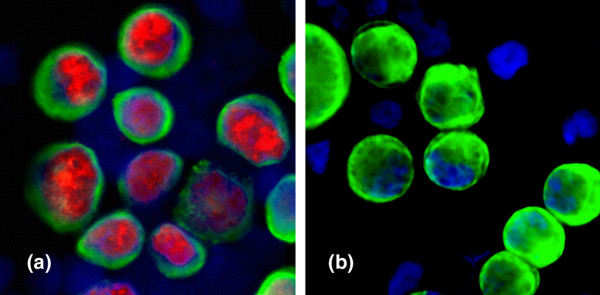
Oestogen receptor (ER) α staining of MCF-7 (positive control) and SKBR3 (negative control) breast cancer cells spiked in bone marrow. A: MCF-7 cancer cells as positive control for ERα-staining. B: SKBR3 cancer cells as negative control ERα-staining.

For establishing the ERα staining procedure, preparations of breast cancer cell lines MCF-7 and SKBR3 mixed with either BM or peripheral blood mononuclear cells (PBMCs) from a healthy volunteer were used (Figure 1). To optimise the staining procedure, all relevant parameters of the protocol were evaluated as follows: types of primary ERα antibodies used were monoclonal mouse antibodies (NCL-L-ER-6F11, Novocastra Laboratories, UK), polyclonal rabbit antibodies (H-184, Santa Cruz Biotechnology, Inc., CA) and monoclonal rabbit antibodies (SP1, Lab Vision, CA); antibody dilutions used were 1:200, 1:100, 1:50 and 1:25 made with DAKO Antibody Diluent (1% BSA in PBS, 0.1% Tween 20); incubation times for primary and secondary antibodies were 30, 45 and 60 minutes; selection of secondary antibodies was with Tex-Red labelled horse anti-mouse AB (Vector Laboratories, Inc., CA), Tex-red labelled goat anti-rabbit AB (CB 11, Biogenex, CA) and Alexa Fluor 594 labelled goat anti rabbit AB (Molecular Probes, Invitrogen, CA); cell fixation was 10 minutes of acetone at 4°C, 100% ethanol for 10 minutes or 0.5% neutral buffered formalin solution for 10 minutes, all three fixations at room temperature. The optimal ERα staining (low background, strong nuclear staining, no cytoplasmic staining) was determined to be as indicated below.

### Immunofluorescence staining of ERα-receptor

After an initial washing step with PBS (Sigma, Munich, Germany), cells were blocked for 30 minutes with normal goat serum (Dako, Glostrup, Denmark) at a 1:10 dilution. The automated double immunofluorescence staining procedure was performed on the DAKO Autostainer using the monoclonal rabbit ERα-antibody SP1 (dilution 1:25, Lab Vision, Fremont, CA, USA) for 60 minutes and secondary detection with a goat anti-rabbit antibody, labelled with Alexa Fluor 594 (1:100, Invitrogen Molecular Probes, Carlsbad, CA, USA) for 30 minutes. Cytospins were then incubated with a pan-cytokeratin (CK) antibody (C11) directly conjugated to fluorescein isothiocyanate (FITC) (1:100, Sigma, Munich, Germany) for 30 minutes. This monoclonal antibody recognises human CKs 4, 5, 6, 8, 10, 13 and 18. Counterstaining was performed with 4'6-diamidino-2-phenylindole (DAPI) in mounting media (Vector Laboratories, Burlingame, CA, USA). Preparations of the breast cancer cell line MCF-7 mixed with PBMCs from a healthy volunteer served as a positive control for CK and ERα staining. ERα negative control slides of SKBR-3/PBMC mixtures were also included with each batch of samples. Cytospins of PBMCs with no added tumour cells served as a negative control for both.

### Fluorescence microscopy

Slides were manually analysed for the presence of tumour cells using a computerised fluorescence microscope Axiophot (×40 oil immersion objectives, Carl Zeiss Micro Imaging GmbH, Göttingen, Germany). To screen for ERα-positive tumour cells, a single-pass filter for individual fluorochromes, FITC, Texas Red or DAPI, and a dual-pass filter for FITC/Texas Red were used. Criteria for evaluation of immunostained cells were based on the criteria of the International Society of Hematotherapy and Graft Engineering Working group for standardisation of tumour cell detection and the consensus statements [10,11]. Criteria for ERα positivity were either moderate or intense staining of the entire nucleus. Slides were evaluated by two, or in doubtful cases three, independent investigators (TF, NK and ES).

### Immunohistochemical staining of the primary tumour

Immunohistochemical analysis was performed either on core biopsies or surgical resection specimens. The tissue was fixed in 4.5% buffered formalin (pH 7.0) and embedded in paraffin. Immunohistochemical staining was performed on 3 to 5 μm thick sections using a commercially available ABC kit (Vectastain, Vector Laboratories, Burlingame, CA, USA). The ERα antibody (clone SP1) was diluted 1:200 in Tris-HCl (pH 7.5) and applied according to the manufacturer's instruction (DCS, Hamburg, Germany). 3,3'diaminobenzidine (DAB) was used as a chromogen. Finally, the slides were counterstained with haematoxylin and mounted for examination. For assessment of the ERα status, the percentage of cells with nuclear reactivity (score 0: none, 1: > 10%, 2: 10 to 50%, 3: 51 to 80%, 4: > 80%) and the intensity of ER staining (score 0: none, 1: weak, 2: moderate, 3: strong) was determined. ERα expression was scored semi-quantitatively using the Remmele-score (score nuclear staining × score intensity of ER staining). Tumours with a score of 2 or more were considered ERα positive.

### Statistical analysis

A chi-squared test or Fisher's exact test was used to evaluate the relation between ERα-positive DTCs and clinicopathological factors. Statistical analysis was performed by SPSS, version 11.5 (SPSS Inc., Chicago, IL, USA). p < 0.05 was considered statistically significant.

## Results

### Patients' charateristics

**Figure 2:**
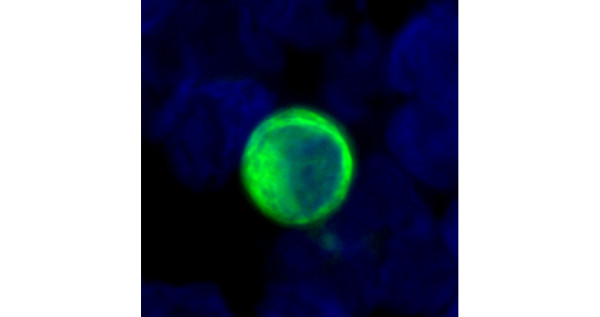
Typical cytomorphology (nuclear size clearly enlarged, high nuclear to cytoplasmic ratio) and immunophenotype (irregular cytoplasmic staining for cytokeratin, cytokeratin filaments can be seen) of a representative disseminated tumour cell from a breast cancer patient. The tumour cell is stained with an anti-cytokeratin-fluorescein isothiocyanate (green) (×40 oil immersion objective).

A total of 254 patients were included in the study. Clinical data are shown in detail in Table 1. Of patients, 82% had ERα-positive primary tumours and DTCs were observed in 107 (42%) of them. Figure 2 shows the cytomorphology and immunophenotype of a representative DTC of a patient with breast cancer. As can be seen, the nuclear to cytoplasmic ratio is high, the nucleus has irregularities and the CK stains the cytoplasm at the periphery of the cell causing a ring-like appearance. These are all accepted morphological criteria for malignant cells. The number of DTCs ranged from 1 to 55 cells/patient (2 × 10^6 ^mononuclear cells). In 38 of the 107 (35%) BM-positive patients, more than one DTC could be detected. No correlation was observed between positive BM status and any of the established prognostic markers including the ERα status of the primary tumour (Table 1).

### ERα expression in disseminated tumour cells

**Figure 3:**
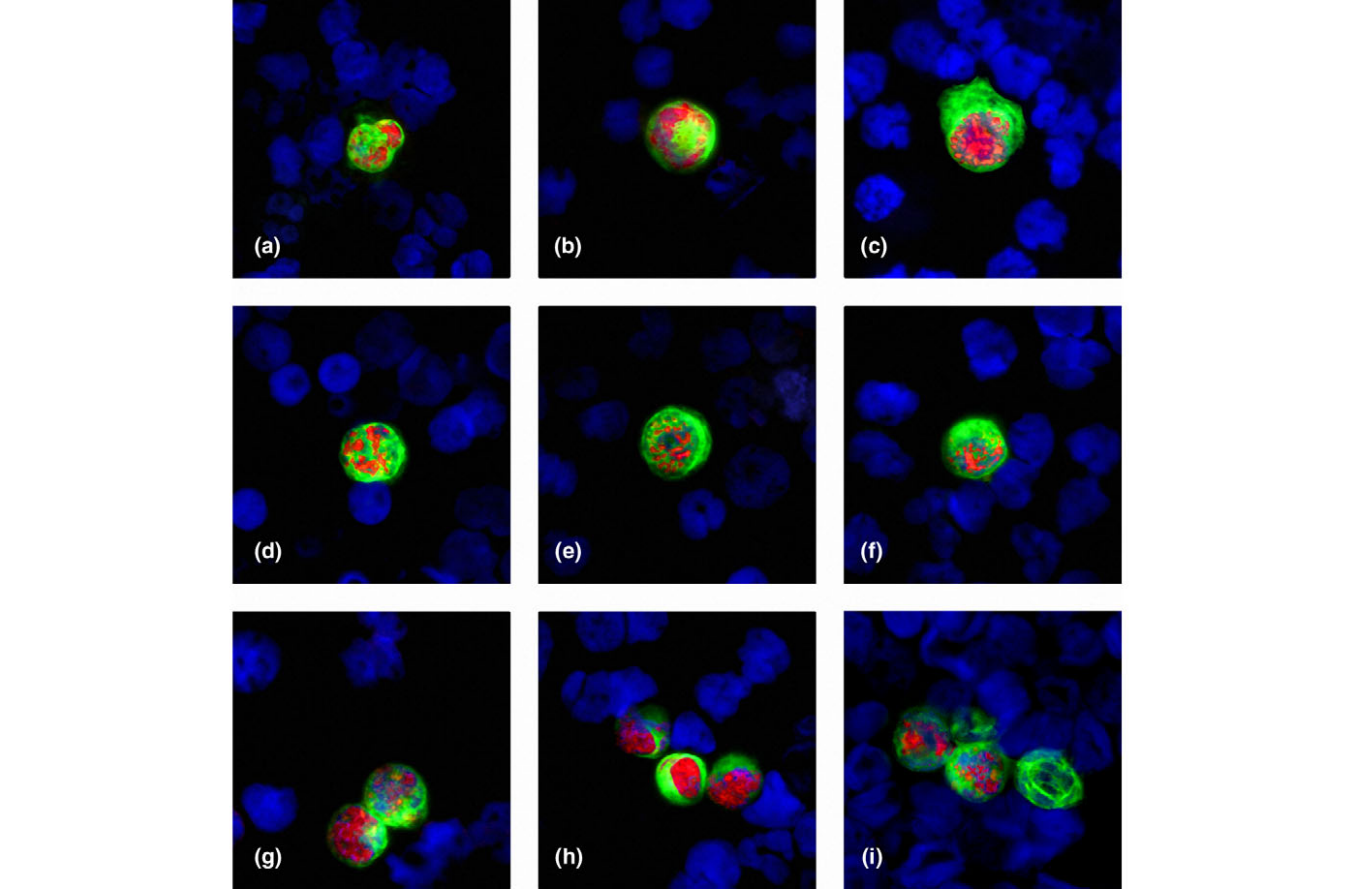
Immunophenotyping of disseminated tumour cells from patients with primary breast cancer. The tumour cells were stained with an anti-cytokeratin-fluorescein isothiocyanate (green) and anti-oestrogen receptor (ER)α detected by a secondary Alexa Fluor 594 labelled goat anti-rabbit antibody (red). Nuclei are stained blue with 4'6-diamidino-2-phenylindole (DAPI) (×40 oil immersion objective). A-F: Breast cancer patients with ERα-positive disseminated tumour cells. G-H: Clusters of ERα-positive disseminated tumour cells. I: Cluster of ERα-positive and ERα-negative tumour cells.

ERα status of DTCs was simultaneously evaluated using a double immunofluorescence staining procedure. The majority of patients (88%) had ERα-negative tumour cells in BM (Table 2). ERα-positive tumour cells could only be detected in 13 of 107 (12%) patients with BM involvement. ERα-positive but CK-negative cells were not observed. Figure 3 shows ERα-positive tumour cells from different patients. As can be seen, the nuclei are strongly stained with the ER antibody.

**Table 2:** Correlation between ERα status of primary tumour and disseminated tumour cells

	ERα status	DTC		Total (%)
			
		ERα negative (%)	ERα positive (%)	
Tumour	ERα negative (%)	18 (17)	1 (1)	19 (18)
	ERα positive (%)	76 (71)	12 (11)	88 (82)

	Total (%)	94 (88)	13 (12)	107 (100)*

*p = 0.8 (chi-squared-test). ER = oestrogen receptor; DTC = disseminated tumour cells.

Of the 107 patients, 38 had more than one DTC in the BM. Of these 38 patients, 28 had only ERα-negative tumour cells (Table 3). Heterogeneity of ERα expression could be detected in the remaining 10 (26%) patients (Figure 3i).

**Table 3:** Correlation between ERα status of primary tumour and heterogeneity of ERα expression in patients with more than one disseminated tumour cell (DTC).

ERα status		DTC			Total (%)
			
		ERα + (%)	ERα – (%)	ERα + (%) & - (%)	
Tumour	ERα – (%)	0	7 (18)	0	7 (18)
	ERα + (%)	0	21 (55)	10 (26)	31 (82)

	Total (%)	0	28 (74)	10 (26)	38 (100)

*p = 0.3 (Fisher-exact-test, two-sided). ER = oestrogen receptor.; + = positive; - = negative.

### Comparison of ERα expression between primary tumour and disseminated tumour cells

The ERα status of the primary tumour could be determined in all 107 patients with detectable DTCs in the BM. ERα positivity of the primary tumour was demonstrated in 88 (82%) of these patients. The concordance rate between ERα status of DTCs and primary tumour was 28%. Only 12 of the 88 (14%) patients with ERα-positive primary tumour had ERα-positive DTCs in the BM. In contrast, 18 of 19 (95%) patients with ERα-negative primary tumours also had ERα-negative DTCs (Table 2). The extent of ERα expression (negative, low, moderate or strong) of the primary tumour was not correlated to the ERα status of DTCs. The comparison of ERα expression between primary tumours and DTCs is summarised in Tables 2 and 3.

## Discussion

Evaluation of ERα status of the primary tumour by immunohistochemistry has been part of routine clinical practice for many years and currently determines patient eligibility for adjuvant endocrine therapy. The assumption is that DTCs will share most characteristics with the primary tumour.

However, an increasing number of publications indicate a more complex relation between the primary tumour and DTCs, with considerable discrepancies noted at the genomic level [12,13]. Supporting this evidence at the phenotypic level are studies looking at HER2 status differences between primary tumours and isolated DTCs [8,14,15].

Similarly, the ERα status of DTCs could be completely different to that of the primary tumour which on the one hand (ERα-negative primary tumour, ERα-positive DTCs) could increase the number of patients eligible for endocrine therapy and on the other hand (ERα-positive primary tumour, ERα-negative DTCs) could explain why endocrine therapy fails in a subset of hormone receptor-positive patients.

Looking at a large patient group, our data confirms findings of previous, smaller studies, indicating that the ERα status of the primary tumour does not necessarily reflect the ERα status of minimal residual disease (Table 4). In an observational study looking at 17 primary tumours and their corresponding DTCs, Ditsch *et al*. found that only two of 11 patients with ERα-positive primary tumours (18%) had ERα-positive DTCs [16]. Reuben *et al*. investigated the ERα status of circulating tumour cells in metastatic breast cancer patients and their corresponding primary tumours: fourteen of 16 patients (88%) had ERα-positive primary tumours, but only three patients had ERα-positive circulating tumour cells [17]. Our results confirm the conclusions that DTCs do not reflect the ERα status of the corresponding primary tumour and a majority of DTCs tend to be ERα negative.

**Table 4:** Comparison of ERα status of the primary tumour and metastatic lesion^§^

Author	*N*	Primary tumour ER + %	ER discordance rate (%)	Site of metastasis	Change ER+/ER- N (%)	Change ER-/ER+ N (%)
Nomura et al. [35]	42	64	10 (24)	LR	10 (24)	0
Kuukasjarvi et al. [25]	50	70	12 (24)	LR, MET	12 (24)	0
Lower et al. [36]	200	58	60 (30)	MET	39 (20)	21 (11)
Li et al. [37]	83	76	24 (29)	LR, MET	83 (16)	11 (13)
Fernandez et al. [27]	26	65	(35)	LN	6 (23)	0
Raemaekers et al. [38]	75	58	14 (19)	LR, LN	8 (10)	6 (8)
Zheng et al. [26]	52	54	(10)	LN	3 (10)	0
Ditsch et al. [16]	17	64	9 (53)	DTC	9 (53)	0
Broom et al. [28]	62	18	11 (18)	DTC, LN, MET	6 (10)	5 (8)
Our study	107	82	77 (82)	BM	76 (71)	1 (1)

^§^distant metastasis, local recurrence, lymph nodes

BM = bone marrow; DTC = disseminated tumour cells; LN = lymph nodes; LR = local recurrence; MET = metastatic lesion.

As mentioned above, these discrepancies between DTCs and the primary tumour are not confined to ERα-expression: Solomayer *et al*. compared the HER2 status of DTC and primary tumour in 137 cases [8] and found that DTCs were more likely to express HER2 than the primary tumour. Meng *et al*. reported HER2-positive circulating tumour cells in nine of 24 (38%) patients with recurrent breast cancer who had HER2-negative tumours [15]. It has been suggested that the high rate of HER2-positive DTCs reflects on their potentially more aggressive phenotype. Other studies looking at markers such as major histocompatibility complex (MHC) III and Ki-76 have reported similar discrepancies [18,19].

Different hypotheses need to be discussed with regard to our findings. One possible explanation is the clonal heterogeneity of the primary tumour: ERα-negative cells could be more likely to disseminate, corresponding to the worse prognosis of predominantly ERα negative tumours and – inversely – to the demonstrated decreased invasiveness and metastatic potential of ERα-expressing breast cancer cells [20,21]. MCF-7 cells, established from a pleural effusion, express ERα and are oestrogen-responsive breast cancer cells. MCF-7 cells do not form metastases in nude mice unless oestrogen supplementation is provided [22-24]. MDA-MB-231 cells were also established from a pleural effusion; however, these cells are ERα-negative and highly invasive. Intravenous injection of MDA-MB-231 cells into the tail vein of nude mice produces tumours [24]. Furthermore, it is well known that about 20 to 30% of patients with ERα-positive primary tumours develop ERα-negative metastatic diseases [25-28].

One interesting hypothesis currently under discussion is the theory that some or all DTCs, the presumed precursor cells of systemic metastatic disease, are in fact cancer stem cells. As recently published, this theory states that tumour growth and formation of secondary tumours can be traced to a small subpopulation of tumour cells, so called cancer stem cells [29,30]. First, most DTCs do not respond to cytotoxic therapy because they are not proliferating and persist over many years in BM. This is also true for tumour stem cells. Secondly, it was also demonstrated that most DTCs in BM were CD44 positive and CD24 low/negative [31]. The CD44-/CD 24-/low phenotype represents a minor population within primary tumours that is associated with self-renewal and tumourigenic potential. In addition, it has been shown that the CD44+/CD24- phenotypes correlated with a higher prevalence of metastases [32]. As breast cancer stem cells have been shown to be generally ERα negative, DTCs with an ERα-negative phenotype despite an ERα-positive primary tumour would agree with the cancer stem cell theory [33,34].

## Conclusion

The phenotypic discrepancies between DTCs and their corresponding primary tumours have the potential to increase our understanding of why treatments are successful in some, but not in other patients, paving the way towards more individualised forms of treatment. The target of adjuvant therapy is the eradication of minimal residual disease. In order to optimise treatment strategies, the phenotypic properties of DTCs – the surrogate marker of minimal residual disease – should be taken into account in addition to characterisation of the primary tumour. Already, the available studies looking at phenotypic properties of DTCs have often found them to be non-proliferative, ERα negative and HER2 positive [8,16,28]. For these patients, expanded treatment with HER2-specific therapies (e.g. trastuzumab and lapatinib) could prove especially beneficial. To further clarify these questions, the next step should be a more generalised and systematic characterisation of DTC-status before and after standard adjuvant therapy for all patients.

## Abbreviations

BM: bone marrow; BSA: bovine serum albumin; CK: cytokeratin; DAB: 3,3' diaminobenzidine; DAPI: 4'6-diamidino-2-phenylindole; DTC: disseminated tumour cells; ER: oestrogen receptor; FITC: fluorescein isothiocyanate; HER2: human epidermal growth factor receptor 2; MHC: major histocompatibility complex; PBMC: peripheral blood mononuclear cells

## Competing interests

The authors declare that they have no competing interests.

## Authors' contributions

TF, GPB, SD, NK, AS and ES made substantial contributions to the conception and design of the study, acquisition of data, and analysis and interpretation of data. TF, SB, HS and HN were involved in drafting the manuscript or revising it. All authors read and approved the final manuscript.
